# miR-200b Inhibits Prostate Cancer EMT, Growth and Metastasis

**DOI:** 10.1371/journal.pone.0083991

**Published:** 2013-12-31

**Authors:** LaTanya V. Williams, Dorina Veliceasa, Elena Vinokour, Olga V. Volpert

**Affiliations:** Department of Urology, Feinberg School of Medicine, Northwestern University, Chicago, Illinois, United States of America; Louisiana State University Health Sciences center, United States of America

## Abstract

miRNA regulate gene expression at post-transcriptional level and fine-tune the key biological processes, including cancer progression. Here, we demonstrate the involvement of miR-200b in the metastatic spread of prostate cancer. We identified miR-200b as a downstream target of androgen receptor and linked its expression to decreased tumorigenicity and metastatic capacity of the prostate cancer cells. Overexpression of miR-200b in PC-3 cells significantly inhibited their proliferation and the formation of subcutaneous tumors. Moreover, in an orthotopic model, miR-200b blocked spontaneous metastasis and angiogenesis by PC-3 cells. This decreased metastatic potential was likely due to the reversal of the epithelial-to-mesenchymal transition, as was evidenced by increased pan-epithelial marker E-cadherin and specific markers of prostate epithelium, cytokeratins 8 and 18. In contrast, mesenchymal markers, fibronectin and vimentin, were significantly downregulated by miR-200b. Our results suggest an important role for miR-200b in prostate cancer progression and indicate its potential utility for prostate cancer therapy.

## Introduction

MicroRNA (miRNA) are short, (21–22 nucleotides) non-coding RNA that bind to mRNA and repress gene expression by mRNA degradation/destabilization or through impaired translation [1]. MicroRNA are first transcribed as 100 bp primary miRNA hairpin structures, which are subsequently cleaved by Drosha into pre-miRNA and exported from the nucleus into the cytoplasm for further processing [2], [3]. Cleavage of pre-miRNA by Dicer proteins yields 22 bp double-stranded molecules, of which one strand is selectively loaded onto the Argonaute proteins, which facilitate miRNA binding to the 3′UTR target sequences on mRNA. MiRNA play pivotal roles in multiple developmental and pathological processes, including cancer of the breast, skin, lung, and cervix [4]–[9].

The miRNA hsa-miR-200b belongs to a family that includes miR-200a, miR-200c, miR-141, and miR-429. Dysregulation of mir-200b has been ascribed a critical role in the epithelial to mesenchymal transition (EMT) and metastasis in cancers such as breast, gastric, and pancreatic carcinomas [10]–[12]. Human miR-200b participates in a double feedback loop with the two transcriptional regulators of E-Cadherin, ZEB1 and ZEB2 [13]. In normal epithelial cells, miR-200b is expressed at high levels; by targeting the 3′UTR regions of pro-metastatic transcriptional factors ZEB1 and ZEB2 it blocks the expression and halts EMT [10]. In contrast, in mesenchymal cells, where ZEB1/ZEB2 expression is abnormally high, they suppress the miRNA of miR-200 family by blocking their promoter activity [13]. Multiple studies assess miR-200b function in EMT, although there are few attempts to address its role in the primary tumor growth.

Here, we demonstrate that miR-200b possesses a similar activity in prostate cancer. Seeking to identify miRNA that contribute to decreased aggressiveness and tumorigenesis in prostate cancer, we performed miRNA profiling of cell lines with inducible expression of androgen receptor previously developed in our lab. We found that miR-200b was significantly upregulated in the poorly tumorigenic PC3 AR-positive cells and that overexpression of miR-200b led to decreased tumor growth. This decreased tumorigenesis was likely due to decreased proliferation. On the other hand, miR-200b strongly upregulated the epithelial cell marker E-cadherin in PCa cells, while the mesenchymal markers Fibronectin and Vimentin were concomitantly decreased. In agreement with the analyses performed in other tumor types, ZEB1, a transcriptional regulator of E-Cadherin was also decreased upon miR-200b overexpression. In addition, miR-200b reduced the invasive potential of the PCa cells *in vitro* and decreased metastasis. Our results show that miR-200b decreases tumor growth and reverses EMT in prostate cancer.

## Methods

### Animal Welfare Assurances

All studies involving laboratory animals (mice) were approved by Northwestern University Animal Care and Use Committee and performed in agreement with the guidelines adopted and suggested by the National Institutes of Health (Animal assurance number A3283-01, expiration date 5/31/2014).

### Cell Lines and Treatment Conditions

PC3 cells transfected with inducible wild-type androgen receptor (AR) or control plasmid were established previously [14]. Cells were maintained in RPMI medium supplemented with 10% Tetracycline-free Fetal Bovine Serum (FBS), 2% penicillin/streptomycin, 50 µg/ml Zeocin and 1 µg/ml Blasticidin. For AR expression, PC3-AR cells were treated for 5 days with 1 µg/ml of Doxycycline and 1 nM of methyltrienolone (R1881) in phenol red free RPMI media supplemented with 10% Charcoal-Stripped FBS, 2% penicillin/streptomycin, 50 µg/ml Zeocin and 1 µg/ml Blasticidin. The parental PC3 cells were maintained in RPMI with 10% FBS and 2% penicillin/streptomycin. All cells were grown at 37°C and 5% CO_2,_ in a humidified incubator.

### Immunoblotting

Cells were plated at a density of 100,000 cells per 10 cm dish. The cells were collected by scraping in phosphate buffered saline (PBS) and centrifuged at 2,500 RPM for 10 minutes at 4°C. The cell pellet was lysed in Ripa buffer (Sigma, St. Louis, MO) supplemented with 1X protease/phosphatase inhibitor solution (Thermo Scientific, Waltham, MA) and centrifuged at 12,000 RPM for 20 minutes at 4°C. The concentration of the supernatant was determined in triplicate by protein assay (DC Protein Assay, Biorad, Hercules, CA). The lysates were electrophoresed on 4%–20% Tris HCL polyacrylamide gels (Biorad, Hercules, CA). Protein lysate was transferred overnight onto PVDF membranes (GE Healthcare Life Sciences, Pittsburg, PA). Each membrane was rinsed in 1X Tris-buffered saline with 0.1% Tween 20 (TBS-T), blocked with 5% non-fat milk in TBS-T and probed with antibodies as indicated in Table S3.

### RNA Extraction

For miRNA detection, total RNA was isolated with miRNeasy Mini kit (Qiagen, Valencia, CA). For mRNA extraction, tumors were snap-frozen in liquid nitrogen and transferred into RNA stabilization solution (Ambion, Life Technologies, Grand Island, NY). The tumors were maintained at −80°C and RNA extraction was performed using miRNeasy Mini kit (Qiagen, Valencia, CA). The RNA concentration and purity was measured with NanoVue Plus spectrophotometer (GE Healthcare Life Sciences, Pittsburg, PA).

### Real Time RT-PCR

RNA was reverse transcribed with miScript II RT Kit (Qiagen, Valencia, CA) following the manufacturer’s instructions. The resulting cDNA was used for real-time PCR analysis using miScript SYBR Green PCR kit (Qiagen, Valencia, CA). For individual miRNA quantification we used miScript Primer Assays (Qiagen, Valencia, CA). For polymerase chain reaction (PCR) analysis, forward and reverse primers were designed and purchased from Integrated DNA Technologies (Coralville, IA) and reactions performed using SYBR Green super mix (Quanta Biosciences, Gaithersburg, MD). The reactions were performed in a Thermal iCycler (Biorad, Hercules, CA). Each sample was tested in triplicate.

### miRNA Array Analysis

PC3-AR cells were treated with Doxycycline (Dox) and R1881 (as described above) for expression and activation of Androgen Receptor (AR), respectively. RNA was extracted as described above and 5 µg of each sample was sent to LC Sciences (Houston, TX) for array analysis. Samples were labeled with Cy_3_ or Cy_5_ and the intensity ratio of each was used to determine the changes in miRNA expression level. A 20-nucleotide RNA control sequence complementary to multiple control probes spotted on each chip was included in each sample as an internal control. The microarrays were performed in triplicate for human, mouse and rat sequences. The potential target spectrum for select miRNA was defined using Sanger miRBase 11.0. Statistical significance was determined using Student’s T-test for p<0.10, p<0.05 and p<0.01.

### miR-200b Overexpression

Shuttle vector encoding precursor sequence hsa-miR-200b (pMIRNA1 pCDH-CMV-MCS-EF1-copRFP) and control vector (pCDH-CMV-MCS-EF1-copRFP) from System Biosciences (SBI, Mountain View, CA) were propagated in Luria Broth +50 µg/ml ampicillin overnight at 30°C in an orbital shaker. Endotoxin-free plasmid DNA was isolated using Endo-Free Maxi Prep kit (Qiagen, Valencia, CA). Lentiviral particles were produced for each plasmid in HEK293T cells using the pMD2.G envelope and psPAX2 2^nd^ generation packaging plasmids (AddGene, Cambridge, MA). The titer was determined by flow cytometry of infected cells as described previously [15]. For transfection, PC3 cells were seeded in 6-well plates at 10^5^ cells/well. The following day, the media was replaced with 1 ml of 1X DMEM containing the appropriate MOI of lentiviral particles for miR-200b and empty vector control. After 6 hr incubation, the cells were fed with RPMI with 10% FBS and 2% penicillin/streptomycin and incubated for an additional 48 hrs. Transduced cells expressing high levels of RFP marker were isolated and flow cytometry combined with cell sorting and maintained in RPMI with 10% FBS, penicillin/streptomycin and 1 µg/ml Puromycin for additional selection.

### Tumorigenesis Assays

#### (1) Subcutaneous inoculation

Parental PC3 cells, PC3 cells transduced with control lentivirus and miR-200b cells were grown as described above, harvested and resuspended at 2×10^7^ cells/ml in serum-free RPMI. One hundred µL of cell suspension (2×10^6^ cells/mouse) were injected subcutaneously into the rear hindquarters of athymic male mice (Nu/Nu, n = 5). The tumors were measured with microcalipers three times a week and the experiment was terminated at 29 days post implantation. The tumors were snap-frozen in liquid nitrogen and stored at −80°C for subsequent analysis.

#### (2) Orthotopic implantation of tumor cells was performed as described previously [16]

Briefly, midline incisions were made, superior to the genital area of supine anesthetized male athymic mice (nu/nu). The bladder was externalized and extended with a cotton swab to reveal the seminal vesicles and the dorsum of the prostate. Tumor cells in 50 µl of serum-free RPMI (10^6^ per animal) were injected into the prostate and the muscle and skin were closed with sutures and metal clips, respectively. All procedures were performed in sterile environment. Metal clips were removed 2 weeks post-injection. Tumor growth was monitored by GFP fluorescence, using small animal imaging system OV100 (Olympus). The experiment was terminated 20 days post implantation. The tumors were excised and snap-frozen for subsequent analysis. To assess metastasis, the residual fluorescence was measured after removal of the primary tumor.

### Proliferation Assay

The cells were seeded in triplicate in 96 well plates at 3×10^3^ cells/well. After 24, 48, and 72 hours, 10 µl of WST-1 reagent (Roche, Indianapolis, IN) was added to each well and incubated for 2 hours. The absorbance was measured for each time point at 450 nm wave length using the Biorad microplate reader (Biorad, Hercules, CA).

### *In Vitro* Invasion Assay

Transwell inserts (Corning, Tewksbury, MA) were coated with 100 µl of 1 mg/ml of Phenol Red free Growth Factor Reduced Matrigel (BD Biosciences, Franklin Lakes, NJ) and allowed to solidify at 37°C in a tissue culture incubator for 24 hours. The cells were resuspended at a density of 10^6^ cells/200 µl in serum-free RPMI, seeded in duplicates in the top part of the coated inserts and incubated at 37°C for 24 hours, with 700 µl of 10% FBS added to the lower chamber as a chemoattractant. The cells were fixed and stained using DiffQuick Fixative solutions (Dade Behring, Malvern, PA) and 6 fields were imaged for each experimental condition. The invasion was calculated as percent invaded cells. The experiment was performed twice.

### Cell Cycle Analysis

The cells were seeded at 10^6^ cells/ml in RPMI supplemented with 0.2% FBS. After 48 hrs the cells were trypsinized, washed twice in cold PBS, fixed in 10 ml 75% ice-cold ethanol and kept overnight at −20°C. The cells were washed twice in 1X PBS at 4°C, resuspended in 2.0 ml DAPI staining solution (0.1% TritonX 100 in 10 ml PBS, 100 µl of 1 mg/ml DAPI) and analyzed by FACS.

### Statistical Analysis

All quantitative data were generated from at least two independent experiments pooled together, with each individual data point performed in triplicates. Standard Deviation (SD) were calculated using Microsoft Excel software. To determine statistical significance of the observed difffrences, we have performed pairwise comparison of the datasets, using one-tailed Student’s T-test. Statistical significance was set at P value not exceeding 0.05.

## Results

### miR-200b is Upregulated by AR

**Figure 1:**
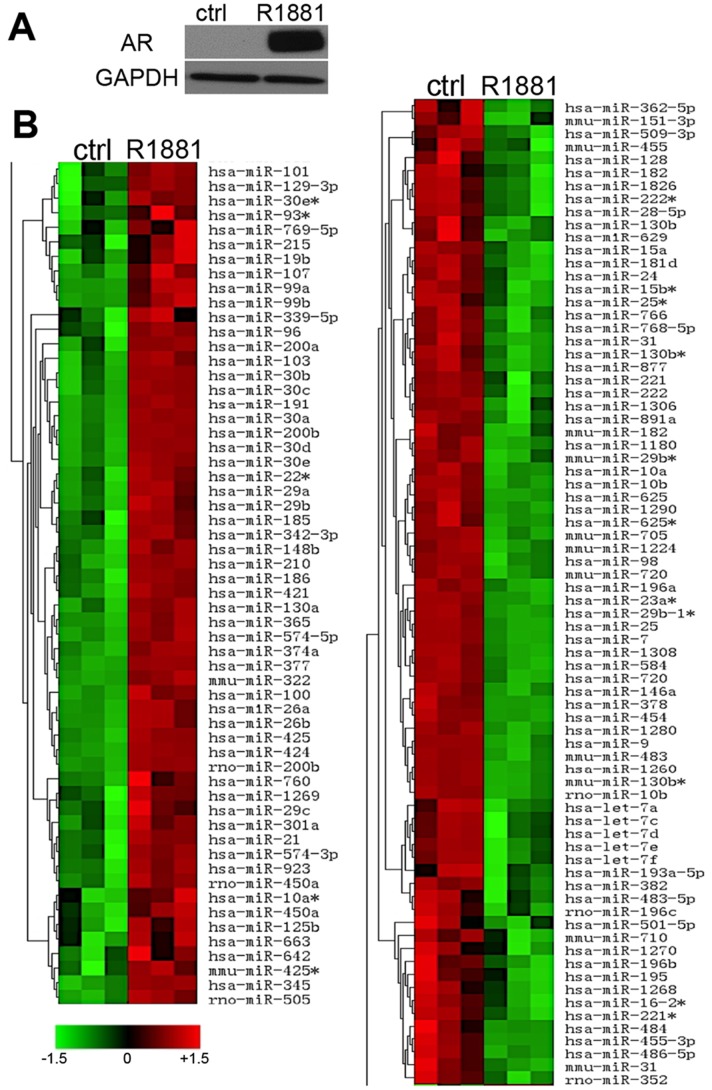
AR activation alters the miRNA profile of PC3-AR cells. (A) Western blot to confirm inducible AR expression in PC3-AR cells. PC3-AR cells were treated 5 days with doxycycline to induce AR expression and with R1881 to induce AR activation and nuclear translocation. The comparison is to untreated control. Total cell lysates were used for analysis. (B). Heat map of miRNA expression in PC3-AR and control cells. Total RNA from cells in A was used for microarray analysis and each sample analyzed in triplicate. The statistical significance for expression changes shown has been determined using Student’s T-test. P values <0.05 were attributed statistical significance.

Previous data from our group showed that androgen receptor (AR) inhibits tumorigenic properties of the AR-negative prostate cancer cell line, PC-3 [14]. MicroRNA are important modifiers of biological functions, including tumor growth. We sought the miRNA that are controlled by AR and involved in the negative regulation of prostate cancer progression. For this purpose, we performed miRNA profiling of the previously generated cell line with tetracycline-inducible AR expression ([14], Fig. 1a). The cells were treated with Doxycycline (Dox) to ensure AR expression and with R1881, to induce AR nuclear translocation and transcriptional activity.

Microarray analysis revealed statistically significant differential expression of 837 individual miRNA in response to AR activation (p<0.05 as determined by Students’ T-test), of which 116 were showed P-values below 0.01 (Figure 1b, Tables S1 and S2). We selected miRNA that were changed more than 2-fold and analyzed their potential targets and functions using Sanger miRBase version 11.0 and Diana Micro T version 3.0. Eight miRNA were selected based on their potential involvement in cancer progression as was determined by *in silico* analysis and published studies. Five of the selected miRNA upregulated in the less tumorigenic PC3-AR cells were also associated with decreased tumorigenesis in other cancers (Table 1). MicroRNA from the miR-200 cluster (miR-200a, miR-200b) have been shown to act as tumor suppressors in gastric, cervical, lung, breast and prostate cancers [9], [10], [17], [18]. Increased miR-200c levels have been linked to decreased metastasis in melanoma, squamous cell carcinoma, and prostate cancer [6], [18], [19]. miR-30a and miR 30-d expression is significantly decreased in chronic myeloid leukemia, non-small cell lung cancer, renal cell carcinoma, and prostate cancer [20]–[23]. In contrast, miR-7 and miR-9 levels were decreased in the less tumorigenic cell line. In accord, miR-7 has been identified as an oncogene in renal cell carcinoma [24] and miR-9 ascribed a role in leukemogenesis [25]–[27]. Low miR-196a levels were observed in malignant melanoma [28] (Table 2).

**Table 1:** miRNA increased with AR expression and inhibitory effect in cancers with increased expression (p<0.05).

miRNA	Fold increase	Cancer type
hsa-miR-200a	13.27	Gastric carcinoma, prostate cancer, cervical cancer
hsa-miR-200b	76.63	Breast cancer, gastric carcinoma, lung adenocarcinoma
hsa-miR-200c	8.94	Melanoma, squamous cell carcinoma, prostate cancer
hsa-miR-30a	4.60	Chronic myeloid leukemia, non-small cell lung cancer
hsa-miR-30d	3.03	Renal cell carcinoma, prostate cancer

**Table 2:** miRNA decreased with AR expression and growth-promoting effect in cancers with increased expression (p<0.05).

miRNA	Fold decrease	Cancer type
hsa-miR-7	8.3	Breast cancer metastasis, glioblastoma
hsa-miR-9	100	gastric carcinoma
hsa-miR-196a	5.2	Melanoma

**Figure 2:**
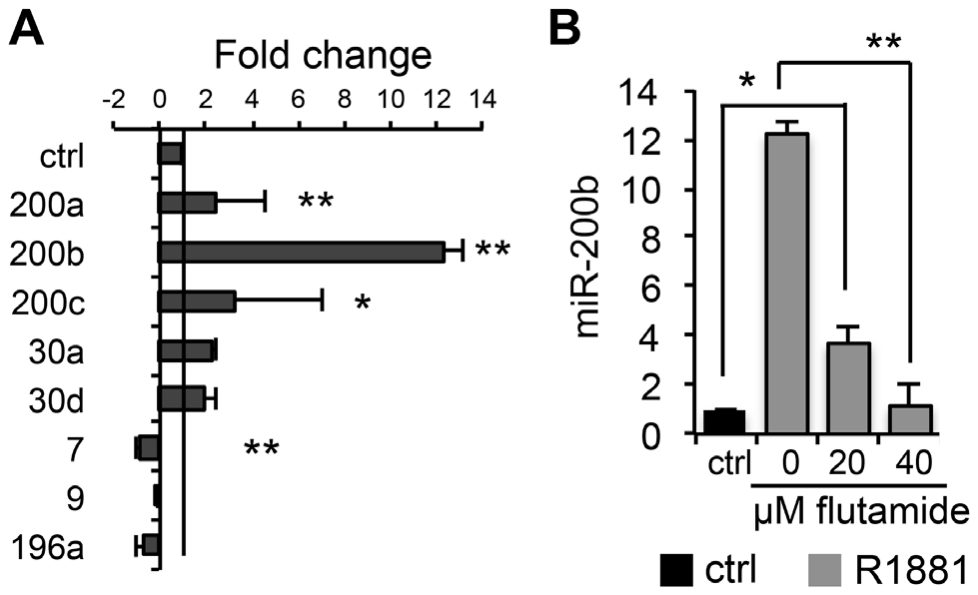
Validation of select miRNA. (A). MiRNA expression was normalized to that of control cells (ctrl). PC3-AR and control cells were treated 5 days with doxycycline to induce AR expression and with R1881 to induce AR activation and nuclear translocation and total RNA collected for analysis. The comparison is to untreated control and RNU1A_1 non-coding RNA is used as an internal control. The statistical significance of observed differences compared to control is * p≤0.05, and **p≤0.01 as was determined by one-tailed Student’s T-test. The average values are calculated for two independent experiments performed in triplicate. (B) AR activation upregulates miR-200b. PC3-AR cells (grey bars) were treated 5 days with both doxycycline to induce AR expression and with R1881 to induce AR activation and nuclear translocation. The comparison is to untreated control. Flutamide was added where indicated to block AR activity. Control (ctrl) PC3-AR cells were left untreated. RNU1A_1 non-coding RNA was used as an internal control. *, p<0.05; **, p<0.01 as determined by Students T-test. The average values were calculated for two independent experiments performed in triplicate.

We have validated select microRNA identified by array analysis using real-time PCR. Indeed, miR-200b, 30a, and 30d were increased, and miR-9 decreased, and these changes were statistically significant (p≤0.05) (Figure 2a). We selected miR-200b for further analysis since it showed the highest fold change and because of its known role in EMT and metastasis [10], [13].

We measured miR-200b levels in PC3-AR cells treated with the combination of Doxycycline, a synthetic androgen R1881 and the anti-androgen flutamide, a competitive inhibitor of AR function. MiR-200b was significantly increased in response to R1881 and this increase was abolished by flutamide in a dose dependent manner, suggesting a direct transcriptional regulation by AR (Figure 2b).

### miR-200b Suppresses the Growth of Prostate Cancer Xenografts

**Figure 3:**
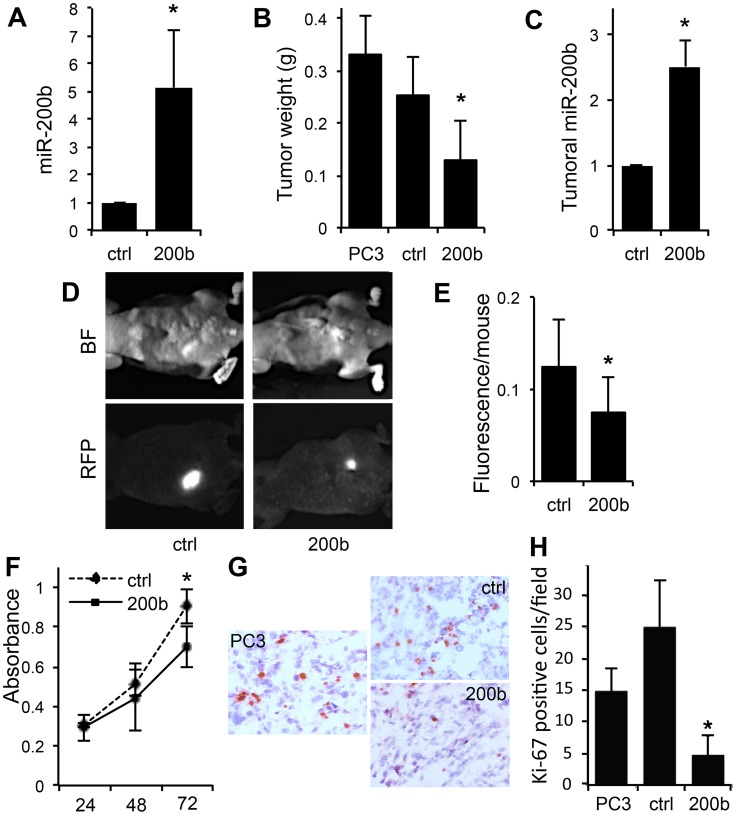
miR-200b is sufficient to decrease tumor growth. A) Forced expression of miR-200b in PC3 cells. PC3 cells were trasduced with a bicistronic lentiviral shuttle vector (pMIRNA1 pCDH-CMV-MCS-EF1-copRFP) encoding hsa-miR-200b and empty vector control (ctrl). Total RNA was isolated and miR- 200b expression measured using real-time RT-PCR. The values represent three independent experiments performed in triplicate. *, p≤0.01. (B) miR-200b reduced tumorigenesis by PC-3 cells. Parental PC3 cells, PC3 cells trasduced with miR control (ctrl) and miR-200b were subcutaneously injected into the rear hindquarters of athymic male mice (n = 5). Tumor weight at day 29 post-injection is shown. *, p≤0.05; **, p≤0.01. (C) The tumors maintained miR-200b expression. Relative miR-200b expression was measured by real-time RT-PCR in the tumors from panel (B). * p≤0.05. (D). miR 200b decreased tumor growth in an orthotopic model of prostate cancer. RFP-tagged PC3-ctrl and PC3-200b cells were implaned in the prostates of athymic male mice. The average fluorescence was measured 20 days post injection. (E) Fluorescence per animal was determined using whole body imaging, with Olympus OV100 system. *, p≤0.05. (F) Cell growth was measured by WST-1 assay. PC3-ctrl or PC3 miR-200b cells were seeded at 3000 cells per well in a 96-well plate. Absorbance was measured at indicated time points using a Biorad Model 680 microplate reader. The results represent the average of three independent experiments performed in triplicate. *, p≤0.05 by Student’s T-test. (G, H) Tumor sections were stained for Ki-67, to evaluate proliferation. Note a significant decrease in Ki-67–positive nuclei in the presence of miR-200b. *, p<0.001.

We next sought the effect of miR-200b expression on prostate cancer tumorigenesis. We overexpressed miR-200b in PC3 cells by transduction with high-titer lentivirus, using empty vector (pCDH-CMV-MCS-EF1-copRFP) as a negative control, and confirmed miR-200b expression by real-time RT-PCR (Figure 3a). The resultant cell lines and parental PC3 cells were subcutaneously injected into male athymic nude mice. Importantly, tumors formed by the cells expressing miR-200b were significantly smaller then the tumors formed by the parental PC3 and the cells transduced with control vector (Figure 3b). Importantly, miR-200b expression was maintained for the duration of the experiment, as was verified by real-time RT-PCR (Figure 3c). Because tumor interactions with its microenvironment play a critical role in tumor progression, we performed orthotopic implantation of the PC3-200b and control cell lines as previously described [16] into the dorsal prostate of male athymic mice. We took advantage of the RFP marker incorporated in the bicistronic lentiviral vector to perform longitudinal *in vivo* imaging. The overall fluorescence pre-dissection was significantly lower in mice bearing PC3 miR-200b positive tumors compared to the vector control group (Figure 3d, e). Thus miR-200b expression was sufficient to reduce tumor growth. Moreover, imaging of abdomen after tumor removal revealed significantly lesser fluorescence due to secondary lesions, suggesting that miR-200b decreased both the primary growth and metastasis by the PC-3 PCa cells (see below).

### miR-200b Suppresses Proliferation of the PCa Cells

Seeking factors that contribute to the decreased tumor growth, we performed cell cycle analysis of PC3 control and PC3 miR- 200b cells and observed a mild increase in G2/M phase om miR-200b positive cells suggesting cell cycle arrest (Figure S1). Indeed, WST-1 assay for viable cells revealed a slight but significant reduction in cell numbers in the miR-200b positive cells as compared to vector control (Figure 3g, h). Combined with significant decrease in Ki-67 positive nuclei in the PC-3 tumors upon miR-200b expression (Figure 3f), our results suggests that the observed decreased in tumor growth was caused by decreased proliferation.

### miR-200b Reverses Epithelial to Mesencymal Transition in PCa Cells

**Figure 4:**
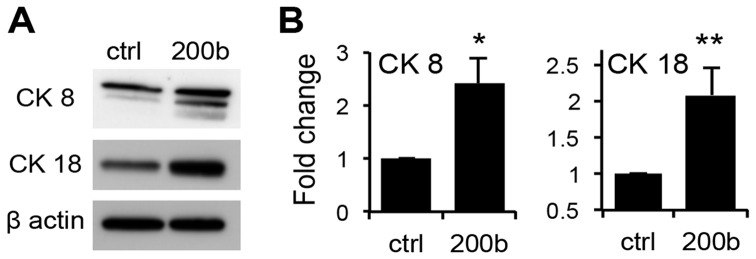
miR-200b promotes differentiation of PC-3 cells. (A). CK 8 and CK 18 were analyzed by western blot of total protein lysates from PC3-ctrl and PC3 miR-200b cells. (B) Quantitative analysis of the experiment (A) with Image J software. The graph represents data average of two independent experiments. *, p≤0.05 and **, p<0.01 by Student’s T-test.

In previous studies, miR-200b expression in breast, gastric, and renal cell carcinoma correlates with the more differentiated state, and re-introduction of miR-200b reverses EMT [10], [11], [29]. These changes can ultimately contribute both to decreased tumor growth and metastasis. Therefore, we analyzed the levels of known EMT and differentiation markers expressed by the PC-3 cells in the presence or absence of miR-200b. First, Western blot analysis showed a significant increase in the typical differentiation markers of prostate epithelium Cytokeratin CK8 and CK18 by miR-200b, suggesting a more differentiated state of miR-200b positive cells (Figure 4).

**Figure 5:**
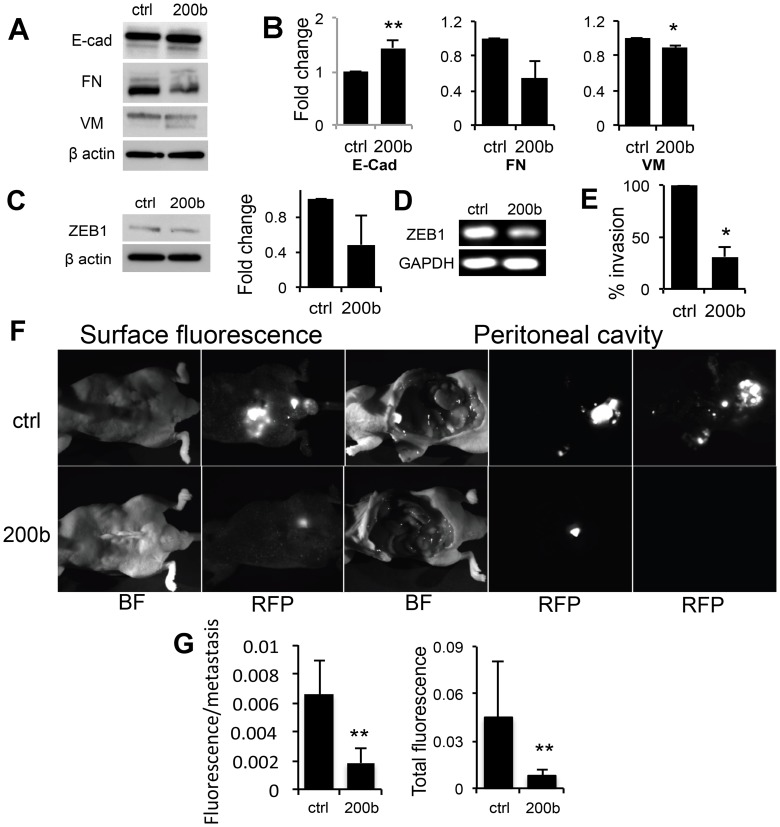
miR-200b reverses EMT and decreases invasion and metastasis by PC3 cells. (A) Markers affected by miR-200b in PC-3 cells. E-cadherin, Fibronectin, and Vimentin were detected in whole cell lysates from PC3 ctrl and PC3 miR-200b cells by western blot. (B) Quantitative analysis of the experiment shown in (A) performed with Image J software. *, p<0.05 and, **, p≤0.01 as determined by Student’s T-test. Two independent experiments were pooled together. (C) Western blot for ZEB1 and quantification performed as above (the average of two experiments is shown). (D) End-point PCR for ZEB1. (E) *In vitro* transwell invasion assay: the comparison of PC3-ctrl and PC3 miR-200b cells. 10% FBS was used as chemoattractant and the experiment was performed in duplicate. *, p<0.05 by Student’s T-test. (F) The *in vivo* spontaneous metastases by PC3-ctrl and PC3 miR-200b cells. The cells were implanted orthotopically in the ventral prostates of male nude mice. The mice were subjected to whole body imaging for RFP-positive masses using Surface image. At the end of experiment, the animals were sacrificed, peritoneal cavity opened and metastasis visualized by fluorescence imaging after the removal of a primary tumor inside the peritoneal cavity and on the frontal wall of the abdomen (Peritoneal cavity). Bright field (BF) and fluorescence (RFP, red fluorescent protein) are shown. (G) Quantification of the experiment shown in (F). The metastases were counted and total fluorescence per metastasis calculated (left). Gross metastatic burden was estimated as total fluorescence per mouse. Ctrl indicates control and 200b indicates miR-200b. **, p≤0.01 by Student’s T-test.

In agreement, the levels of E-cadherin were also increased (Figure 5a), suggesting a shift towards epithelial phenotype. Furthermore, Vimentin, a mesenchymal marker, underwent a statistically significant decrease upon miR-200b expression. Another mesenchymal marker, fibronectin (FN) displayed a similar trend although the decrease did not reach statistical significance (Figure 5b). Because in previous studies miR-200b controlled E-Cadherin and EMT by repression of the transcriptional factor ZEB1, we have assessed both total ZEB1 protein and RNA levels in miR-200b-positive and control PC-3 cells. We observed a significant and reproducible decrease in ZEB1 protein and mRNA in response to miR-200b (Figure 5c, d). This result is consistent with the increased E-cadherin expression (Figure 5a). Our results clearly demonstrate that miR-200b expression promotes the epithelial cell characteristics and suppresses mesenchymal features in PC3 cells.

### miR-200b Decreases Invasion and Metastasis by PC-3 Cells

Because miR-200b caused EMT reversal in PCa PC-3 cells, it was reasonable to expect a decreased invasive potential as well. Using a standard procedure to measure cell invasion, we observed a significant decrease in the percentage of invaded cells due to miR-200b expression as compared to control (Figure 5e). This decreased invasion likely underlies the decreased regional metastasis by PC3 miR-200b cells *in vivo*.

Orthotopic injection of parental PC-3 and vector control cells into the dorsal prostates of athymic male mice resulted in spontaneous metastasis, as was detected after the removal of the primary tumors (Fig. 5f and Figure S2). The overall fluorescence due to metastasis was dramatically decreased in mice implanted with the PC-3 cells overexpressing miR-200b (Figure 5f, g and Figure S2). Thus our data suggest that miR-200b significantly decreases mesenchymal features in prostate cancer cells and thus reduces their invasive characteristics and metastatic potential.

### miR-200b Reduces Tumor Angiogenesis

**Figure 6:**
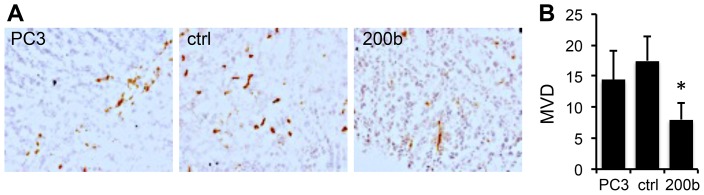
miR-200b suppresses angiogenesis in PCa. Sections from the tumors formed by control and miR-200b positive PC-3 tumors were stained for the endothelial marker CD31 and the number of microvessels per field determined in ten 10× fields. *, P<0.01.

Another feature that may enable metastasis is angiogenesis and EMT is often accompanied by increased angiogenesis. The expression of miR-200b appeared to reverse angiogenic switch in PC-3 cells as was evidenced by decreased microvascular density in the miR-200b positive tumors compared to the parental PC-3 or vector control tumors (Fig. 6).

## Discussion

The miR-200 family is a tumor-suppressive family of microRNA that play critical roles in suppression of EMT. While the role of miR-200 family in breast and some other epithelial cancers is well established, there are few findings linking miR-200 with prostate cancer. A small-scale study (20 patients) suggests an association between the biochemical relapse after radical prostatectomy and the lower levels of miR-200c [30]. One study by He *et al.* demonstrates that low levels of miR-200b-3p in androgen-independent cells are caused by decreased expression of the p-53-related protein p73 and contribute to increased proliferation [31]. The two clusters encoding miR-200 family are found on chromosomes 1 and 12, with miR-200b, miR-200a, and miR-429 on chromosome 1 and miR-200c and miR-141 on chromosome 12. MicroRNA 200c is thought to be epithelial-specific and its expression is repressed by hypermethylation of the proximal CpG island in the fibroblasts and in breast cancer cell lines [32]. In the PC-3, but not in LnCaP and DU145 PCa cells, similar hypermethylation of the CpG island coincides with the low levels of miR-200c and miR-141 [32]. Consistent with our results, in this study the PC3 cells negative for miR-200c and miR-141, display a clear mesenchymal phenotype and the capacity for invasion and metastasis [32]. Two studies indicate the role for miR-200 in tumorigenesis by the PCa stem cells. In stem-like PCa cells the expression of Sox2, Nanog, Oct4, Lin28B and/or Notch1 is consistent with enhanced clonogenic survival and the ability to form spheroids (prostaspheres). These stem-like features are connected to decreased expression of miR-200b/c and/or of let-7 family whereby re-expression of miR-200 inhibits prostasphere formation and reduces Notch1 and Lin28B, the drivers of self-renewal [33]. In an unrelated study, the invasive tumorigenic clones derived from the benign prostatic hyperplasia (BPH)-1 cell line chronically exposed to TGF-β display prominent EMT features with high levels of SNAI2, ZEB1, and ZEB2, all confirmed miR-200 targets; however, the basal miR-200 levels in these cells remain unaltered suggesting a divergent mechanism [34].

The augmented stem-like features suggest enhanced tumorigenicity. Indeed, multiple groups have shown that miR-200b alters the growth rate of experimental tumors by the cultured PCa cells [35]. Our data clearly corroborate these observations. MiR-200b expression dramatically reduced the size of both subcutaneous and orthotopic tumors formed by the PC-3 cells. In contrast with previous observations in breast cancer cells and preclinical models of breast cancer, which showed dramatic reduction of proliferative capacity by miR-200 family members [36], we found that miR-200 only modestly decelerated the growth of PC-3 cells *in vitro* and *in vivo*. It is possible that miR-200b alone is insufficient to inhibit proliferation: microRNA are generally changed in concert and multiple miRNA target a single trait. The miR-200 family has often been studied in tandem, and our array analysis also revealed lesser but significant increases in miR-200c and miR-429, which could further enhance anti-proliferative effect of miR-200b.

Our study for the first time identified miR-200b as direct target of androgen receptor: in AR-expressing PC-3 cells miR-200 levels were increased by DHT and the anti-androgen Flutamide fully abolished this increase. In PCa, hyperactivation or amplification of AR signaling often increases proliferation and contribute to tumorigenesis [37]. However, in some contexts, including PC-3 cells, AR opposes PCa progression [14], [38]–[40]. This tumor suppressive activity is consistent with AR role as the driver of differentiation in prostate epithelium [41]. Our results suggest that the AR may contribute to the maintenance of miR-200 family members in the normal poorly tumorigenic transformed prostate epithelium and that this pathway is disrupted in the aggressive, metastatic PCa. In our previous studies AR expression in PC3 cells led to G1 cell cycle arrest and senescence. Despite its effect on cell proliferation, miR-200b failed to cause growth arrest or senescence *in vitro* and *in vivo* (data not shown) suggesting that AR-induced senescence occurs independently of miR-200b. Interestingly, in the study by He et al., decreased miR-200b occurred in the cells with perturbed AR signaling (PC-3 and androgen-independent sub-clone of LNCaP; however, this study established a causative link between miR-200b expression and p73 (see above) but not AR [31].

The role of the miR-200 family in the suppression of mesenchymal characteristics and metastasis is firmly established in kidney, gastric, and breast cancers [11]. Similarly, the loss of miR-200 is associated with EMT of the PCa. In murine adenocarcinoma of the prostate there is a direct self-reinforcing regulatory loop where miR-1 and miR-200 are decreased in the course of progression causing de-repression of Slug (SNAI2), which in turn blocks miR-1 and miR-200 transcription and amplifies EMT [42]. In addition, the exposure to exogenous PDGF-B as well as its stable overexpression dramatically augments the EMT features in cultured PC-3 PCa cells. In this case, the enhanced mesenchymal features are associated with a decrease of miR-200b and re-introduction of miR-200b significantly inhibits the migration and invasion due to PDGF-B [35]. In agreement with these findings, miR-200b increased epithelial features of the PC-3 cells and reduced some of the mesenchymal markers, such as vimentin and fibronectin. The expression levels of CK8 and CK18, the markers of AR-positive, fully differentiated prostatic epithelium [43], [44], were much higher in the presence of miR-200b.

In agreement, we found the partial reversal of the EMT signature upon miR-200b expression, with increased E-cadherin and decreased Fibronectin, Vimentin, and ZEB1. These changes could be more dramatic if multiple miR-200 family members were activated, however even a single member, miR-200b, caused a dramatic reduction in the invasive potential of the PC-3 cells and a striking decrease in spontaneous regional metastasis in an orthotopic model. We were unable to demonstrate the change in SNAI2, which may reflect the difference in the target spectrum between the murine and human cells.

The effect of miR-200 family on angiogenesis has been established only recently. One study implicates miR-200c in the regulation of angiogenesis in the endometrial tissue, by suppressing vascular endothelial growth factor (VEGF) and its VEGF receptor 2 (FLT1) [45]. Another very recent analysis elucidates the link between increased angiogenesis in the breast, ovarian and renal cancers and the loss of miR-200a and b, whereby miR-200a/b collectively block the expression of interleukin-8 (IL-8) and CXCL1, a small cytokine previously called GRO1 [46]. The IL-8 contributes the angiogenesis proper (the sprouting of pre-existing capillaries), while CXCL1 supports the recruitment of the bone marrow-derived progenitor cells to the tumor neovasculature. In agreement we observe a marked decrease in angiogenesis in prostate cancer xenografts upon miR-200b overexpression. This decreased angiogenesis is likely to further attenuate metastatic burden in a model where PC-3 cells metastasize spontaneously from an orthotopic site.

In conclusion, we identified miR-200b as a factor in decreased tumor growth when expressed in PC3 cells. We also found evidence that miR-200b could reverse or hinder EMT as described in other cell lines and for the first time demonstrated the anti-metastatic activity of miR-200 in an orthotopic preclinical model of PCa that recapitulates PCa progression in an appropriate microenvironment, which includes microvasculature. The inclusion of other members of the miR-200 family may shed further light on their role in PCa proliferation and survival and provide compelling rationale for miR-200 based treatment for the metastatic PCa.

## Supporting Information

Figure S1 (PDF)

Figure S2 (PDF)

Table S1 (PDF)

Table S2 (PDF)

Table S3 (PDF)
