# Supplementary material for: miR-200b Inhibits Prostate Cancer EMT, Growth and Metastasis
Source: PLoS One. 2013 Dec 31;8(12):e83991. doi: 10.1371/journal.pone.0083991 (PMC3877136; doi:10.1371/journal.pone.0083991)
Supplement: Figure S1 — (PDF) [file pone.0083991.s001.pdf]

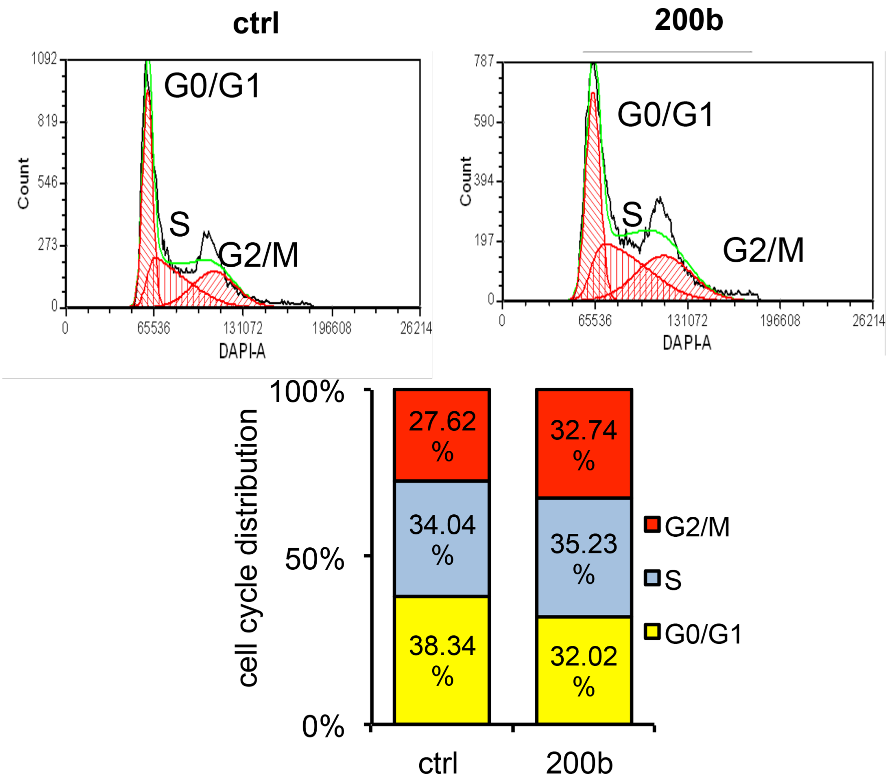

Supplemental Figure 1. miR-200b increases the number of cells in G2M phase arrest. FACS was used to determine cell cycle distribution in control and miR-200b positive PC-3 cells. DNA content was determined by DAPI staining and the experiment was performed in triplicate.
