# Supplementary material for: miR-200b Inhibits Prostate Cancer EMT, Growth and Metastasis
Source: PLoS One. 2013 Dec 31;8(12):e83991. doi: 10.1371/journal.pone.0083991 (PMC3877136; doi:10.1371/journal.pone.0083991)
Supplement: Figure S2 — (PDF) [file pone.0083991.s002.pdf]

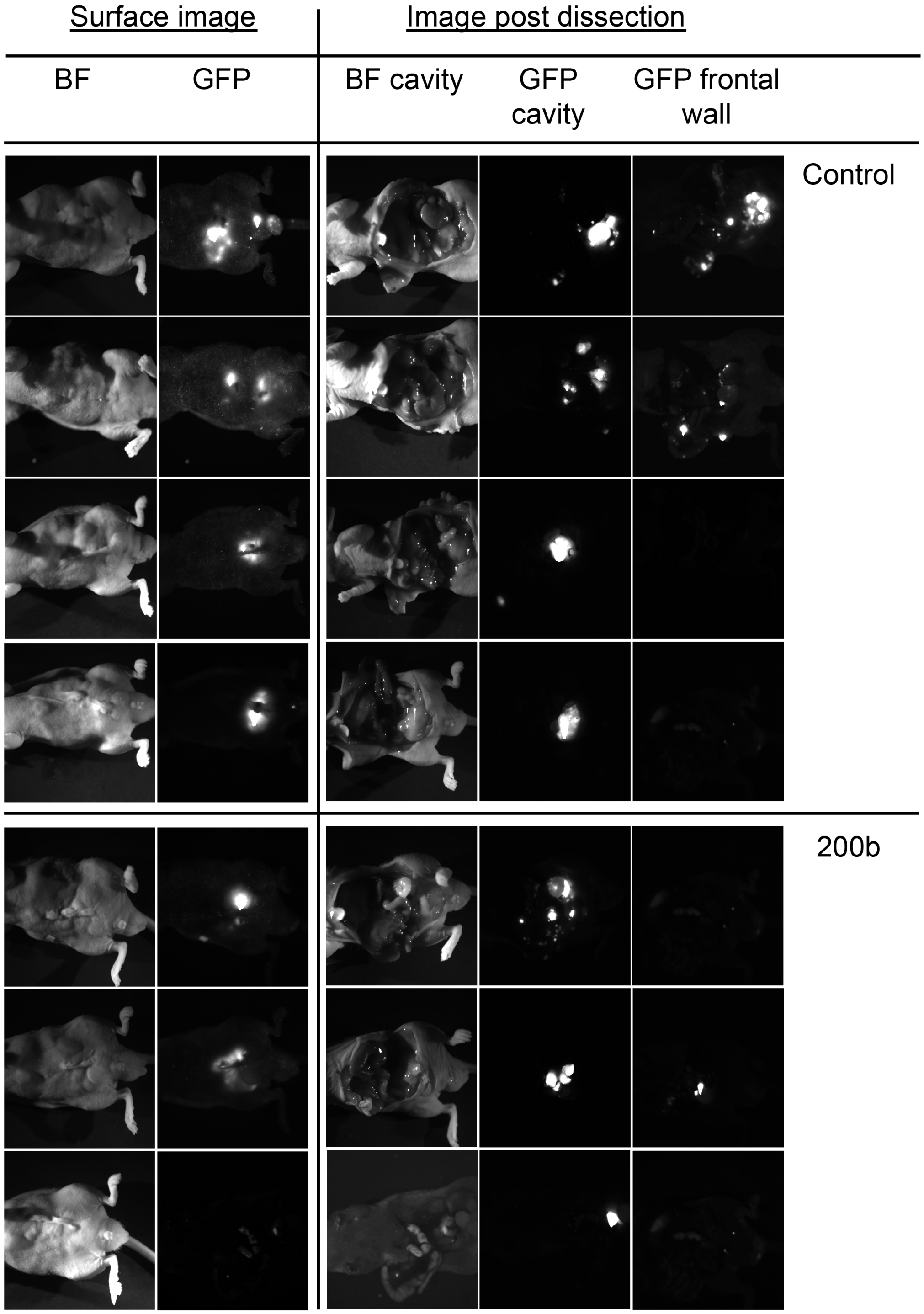

Supplementary Figure S2. The effect of miR-200b on spontaneous metastasis. Male nude mice bearing orthoropic tumors formed by PC-3 cells transduced with lentiviral miR-200b or control vector were subjected to fluorescence imaging using OV100 system (Olympus). The images were taken prior to dissection (left) and post dissection (right), after the removal of primary tumor, to evaluate metastasis to the peritoneal cavity. The images of the cavity or the inner side of the frontal wall of peritoneum are shown.
