# Supplementary material for: miR-200b Inhibits Prostate Cancer EMT, Growth and Metastasis
Source: PLoS One. 2013 Dec 31;8(12):e83991. doi: 10.1371/journal.pone.0083991 (PMC3877136; doi:10.1371/journal.pone.0083991)
Supplement: Table S1 — (PDF) [file pone.0083991.s003.pdf]

Supplementary Table S1. miRNA increased with Androgen Receptor Expression p<0.01

miRNA	log2 AR+/ctrl
hsa-miR-200b	6.26
hsa-miR-95	4.92
hsa-miR-200a	3.73
hsa-miR-200c	3.16
hsa-miR-201	3.14
hsa-miR-335	3.00
hsa-miR-186	2.98
hsa-miR-429	2.95
hsa-miR-30e	2.48
hsa-miR-22*	2.47
hsa-miR-129-3p	2.40
hsa-miR-574-3p	2.34
hsa-miR-96	2.22
hsa-miR-30a	2.20
hsa-miR-424	2.14
hsa-miR-200b*	2.05
hsa-miR-22	1.94
hsa-mir-30e*	1.78
hsa-miR-1269	1.64
hsa-miR-30d	1.60
hsa-miR-191	1.55
hsa-miR-425	1.44
hsa-miR-450a	1.27
hsa-miR-342-3p	1.14
hsa-miR-29b	1.14
hsa-miR-760	1.13
hsa-miR-301a	1.05
hsa-miR-923	1.00
hsa-miR-192	0.93
hsa-miR-126	0.92
hsa-miR-19b	0.86
hsa-miR-30b	0.85
hsa-miR-20-3p	0.85
hsa-miR-30c	0.84
hsa-miR-148b	0.81
hsa-miR-26b	0.80
hsa-miR-21	0.76
hsa-miR-26a	0.73
hsa-miR-574-5p	0.70
hsa-miR-421	0.66
hsa-miR-132	0.65
hsa-miR-374a	0.61
hsa-miR-424*	0.59
hsa-miR-29a	0.56
hsa-miR-107	0.52
hsa-miR-130a	0.50
hsa-miR-103	0.47
hsa-miR-99a	0.47
hsa-miR-100	0.42
hsa-miR-29c	0.34
hsa-miR-99b	0.27
hsa-miR-361-5p	0.20
hsa-miR-106a	0.20
hsa-miR-17	0.19
