# Supplementary material for: miR-200b Inhibits Prostate Cancer EMT, Growth and Metastasis
Source: PLoS One. 2013 Dec 31;8(12):e83991. doi: 10.1371/journal.pone.0083991 (PMC3877136; doi:10.1371/journal.pone.0083991)
Supplement: Table S2 — (PDF) [file pone.0083991.s004.pdf]

Supplementary Table S2. miRNA decreased with Androgen Receptor Expression p<0.01

miRNA	log2 AR+/ctrl
hsa-miR-9	-6.15
hsa-miR-7	-3.08
hsa-miR-891a	-2.82
hsa-miR-1260	-2.78
hsa-miR-196a	-2.36
hsa-miR-23a*	-2.21
hsa-miR-584	-1.88
hsa-miR-625	-1.82
hsa-miR-485-5p	-1.74
hsa-miR-130b*	-1.70
hsa-miR-625*	-1.67
hsa-miR-29b-1*	-1.63
hsa-miR-1308	-1.36
hsa-miR-146a	-1.36
hsa-miR-196b	-1.35
hsa-miR-720	-1.35
hsa-miR-454	-1.26
hsa-miR-31	-1.23
hsa-miR-629	-1.22
hsa-miR-15a	-1.12
hsa-miR-15b*	-0.96
hsa-miR-1290	-0.96
hsa-miR-10b	-0.91
hsa-miR-1280	-0.90
hsa-miR-221*	-0.89
hsa-miR-877	-0.88
hsa-miR-25	-0.84
hsa-miR-195	-0.76
hsa-miR-1826	-0.74
hsa-miR-1268	-0.73
hsa-miR-181d	-0.73
hsa-miR-93	-0.63
hsa-miR-28-5p	-0.59
hsa-miR-455-3p	-0.58
hsa-let-7e	-0.54
hsa-miR-98	-0.53
hsa-miR-10a	-0.53
hsa-miR-193a-5p	-0.51
hsa-let-7f	-0.50
hsa-miR-182	-0.45
hsa-let-7d	-0.40
hsa-let-7c	-0.37
hsa-let-7a	-0.35
hsa-miR-222	-0.32
hsa-miR-221	-0.20
hsa-let-7b	-0.18
hsa-miR-24	-0.17
hsa-miR-15b*	-0.13

\
