# Supplementary material for: miR-200b Inhibits Prostate Cancer EMT, Growth and Metastasis
Source: PLoS One. 2013 Dec 31;8(12):e83991. doi: 10.1371/journal.pone.0083991 (PMC3877136; doi:10.1371/journal.pone.0083991)
Supplement: Table S3 — (PDF) [file pone.0083991.s005.pdf]

Supplementary Table S3. Antibodies with dilution factors

**Antibody**	**Supplier**	**Number**	**Dilution**
Androgen Receptor	Santa Cruz	sc-816	1:500
Cytokeratin 8	Santa Cruz	sc-52354	1:200
Cytokeratin 18	Santa Cruz	sc-sc-58727	1:200
E-cadherin	Cell Signaling	3195	1:1000
Fibronectin	Santa Cruz	sc-9068	1:500
Vimentin	Cell Signaling	5741	1:2000
ZEB1	Cell Signaling	3396	1:500
β actin	Sigma	A5441	1:1000
GAPDH	Sigma	G9295	1:20,000
